# Data on the optimization of the formula of *Xiaokeyinshui* extract combination treating diabetes mellitus using uniform experimental design in mice

**DOI:** 10.1016/j.dib.2020.106134

**Published:** 2020-08-05

**Authors:** Jiewen Zhou, Jun Pan, Zhinan Xiang, Qiuyan Wang, Qilin Tong, Jinbo Fang, Luosheng Wan, Jiachun Chen

**Affiliations:** Hubei Key Laboratory of Natural Medicinal Chemistry and Resource Evaluation, College of Pharmacy, Huazhong University of Science and Technology, Hangkong Road 13, Wuhan 430030, Hubei Province, China

**Keywords:** Traditional Chinese medicine, *Xiaokeyinshui* extract combination, Diabetes mellitus, Formula optimization, Uniform experimental design

## Abstract

This dataset is supplementary to our accepted article in Journal of Ethnopharmacology [1]*. Xiaokeyinshui* (XKYS) formula, an anti-diabetic formula, was recorded in many ancient Chinese medical books. *Xiaokeyinshui* extract combination (XEC) originated from this ancient formula, consisting extracts of four herbal drugs, i.e., Coptidis Rhizoma, Liriopes Radix, bitter melon, and Cassiae Semen. In this study, herb extracts were prepared and mixed, producing *Xiaokeyinshui* extract combination (XEC). The optimized formula of XEC was also investigated via uniform experimental design. Diabetes was induced in Kunming mice, using high-sugar-high-fat diet combined with injection of streptozotocin (STZ) intraperitoneally. Different formulae of XEC were intragastrically administered to diabetic mice for 28 days. Fasting blood glucose (FBG), oral glucose tolerance test (OGTT), hemoglobin A1c (HbA1c), total cholesterol (TC), total triglyceride (TG) were measured to assess the anti-diabetic effects of each formula. Multivariate second degree polynomial model was applied in the fitting of metabolic parameters, and the extremum value of each regression model was calculated using grid algorithm. In addition, an optimized formula of XEC was subjected to validation experiment in mice model. This data could provide basis for a reasonable analysis for the optimization of the formula of XEC.

Specifications Table

**Table:** 

Subject	Pharmacology
Specific subject area	Complementary and Alternative Medicine; Endocrinology, Diabetes and Metabolism
Type of data	Table
How data were acquired	This data was acquired from 96 male Kunming mice categorized into 12 groups in the experiment of uniform design and 32 male Kunming mice categorized into 4 groups in the validation experiment.
Data format	Raw Analyzed
Parameters for data collection	Blood glucose (BG), hemoglobin A1c (HbA1c), total cholesterol (TC), total triglyceride (TG)
Description of data collection	The level of BG were measured using plasma glucose test strips. HbA1c measurement was conducted via Ultra2 GHb meter. Analysis of TC and TG was performed using commercial kits.
Data source location	Wuhan, China
Data accessibility	With the article.
Related research article	Jiewen Zhou, Jun Pan, Zhinan Xiang, Qiuyan Wang, Qilin Tong, Jinbo Fang, Luosheng Wan, Jiachun, Chen. *Xiaokeyinshui* extract combination, a berberine-containing agent, exerts anti-diabetic and renal protective effects on rats in multi-target mechanisms. J Ethnopharmacol. 2020, 113098. https://doi.org/10.1016/j.jep.2020.113098

Value of the Data

• This data presented here not only describes the design of different formulae of *Xiaokeyinshui* extract combination (XEC) using uniform experimental design, but also describes the effects of different formulae of XEC on diabetic mice, in which diabetes was induced with a combination of both high-sugar-high-fat diet and injection of streptozotocin.
• This data presented here provides a series of regression equations using the multivariate second degree polynomial model, which provides basis on the optimization on the formula of XEC.
• This data can be useful for the study design aiming to decipher the mechanisms of action of XEC in the future.
• This data can be useful for researchers on traditional Chinese medicine, especially for those who focus on the design of traditional Chinese medicine formula.

## 1 Data description

The data were analyzed with IBM SPSS Statistics V22.0. Results were presented as means ± standard deviations (SD). T-test was applied in the assessment of the differences among multiple groups, with *p* < 0.05 as statistically significant.

Table 1 provided a uniform design table U_9_(9^4^), being the principle in the experimental design. Table 2 presented the daily dose of herb extracts for mouse in each formula of *Xiaokeyinshui* extract combination (XEC).

**Table 1:** Uniform experimental design table U_9_(9^4^).

Levels	Factors			
	X_1_	X_2_	X_3_	X_4_
1	1	2	4	7
2	2	4	8	5
3	3	6	3	3
4	4	8	7	1
5	5	1	2	8
6	6	3	6	6
7	7	5	1	4
8	8	7	5	2
9	9	9	9	9

**Table 2:** Daily dose of herb extracts for mouse (mg/kg/d).

Formula	TACR (X_1_)	LRP (X_2_)	BME (X_3_)	CSE (X_4_)	Total daily dose
XEC1	16	130	59	56	261
XEC2	31	270	123	40	464
XEC3	46	410	43	24	523
XEC4	61	550	107	8	726
XEC5	76	60	27	64	227
XEC6	91	200	91	48	430
XEC7	106	340	11	32	489
XEC8	121	480	75	16	692
XEC9	136	620	139	72	967

Table 3 presented effects of different XEC formulae on fasting blood glucose (FBG) in mice. Table 4 presented effects of different XEC formulae on blood glucose (BG) levels in 2 h oral glucose tolerance test (OGTT) in mice. Table 5 presented effects of different XEC formulae on hemoglobin A1c (HbA1c), total cholesterol (TC), total triglyceride (TG) in mice. In brief, Tables 3–5 presented metabolic parameters of mice. Raw data relating to these parameters could be seen in Supplementary Materials, Tables S1–S4.

**Table 3:** Effects of different XEC formulae on FBG (*n* = 8).

Group	FBG (mmol/L)					Change of FBG (%)
	Day 0	Day 7	Day 14	Day 21	Day 28	
NC	3.79 ± 0.48⁎⁎	3.94 ± 0.42⁎⁎	3.20 ± 0.52⁎⁎	3.13 ± 0.51⁎⁎	3.59 ± 0.49⁎⁎	–
DC	15.53 ± 1.39	16.23 ± 1.72	15.90 ± 2.04	15.70 ± 3.00	15.75 ± 1.21	−1.42
MET	15.48 ± 2.36	13.46 ± 1.40⁎⁎	11.31 ± 1.18⁎⁎	10.10 ± 0.99⁎⁎	8.69 ± 2.13⁎⁎	43.86
XEC1	15.01 ± 1.91	16.13 ± 1.82	15.69 ± 2.66	14.81 ± 2.01	13.89 ± 2.26	7.46
XEC2	15.59 ± 1.63	16.51 ± 1.29	14.76 ± 2.74	14.06 ± 2.39	13.20 ± 2.26*	15.33
XEC3	16.45 ± 2.83	18.35 ± 2.50	17.19 ± 1.67	15.84 ± 2.95	14.90 ± 1.69	9.42
XEC4	15.66 ± 2.91	18.71 ± 1.30	18.19 ± 2.68	17.69 ± 3.38	17.85 ± 2.84	-13.98
XEC5	17.64 ± 2.89	17.99 ± 2.54	16.93 ± 2.82	16.60 ± 1.75	15.86 ± 1.19	10.09
XEC6	16.35 ± 2.00	15.81 ± 1.38	13.00 ± 2.42*	12.48 ± 1.27*	9.68 ± 1.48⁎⁎	40.80
XEC7	16.50 ± 2.26	17.30 ± 1.51	15.55 ± 2.28	14.60 ± 1.44	13.85 ± 1.93*	16.06
XEC8	15.83 ± 3.28	17.56 ± 2.38	16.73 ± 1.77	17.39 ± 2.29	16.83 ± 3.55	-6.12
XEC9	15.59 ± 3.44	17.93 ± 1.92	20.16 ± 2.86	20.74 ± 3.85	22.61 ± 5.01	-45.03

The data were presented as means ± SD. T-test was applied in the assessment of the differences among multiple groups.

⁎⁎ *p*<0.01,

⁎ 0.01≤*p*<0.05, versus DC.

**Table 4:** Effects of different XEC formulae on BG levels in 2h OGTT (*n* = 6).

Group	BG (mmol/L)				AUC (mmol/L•h)
	0 h	0.5 h	1 h	2 h	
NC	3.48 ± 0.31⁎⁎	10.08 ± 1.54⁎⁎	7.42 ± 0.66⁎⁎	5.60 ± 1.01⁎⁎	14.28 ± 0.88⁎⁎
DC	16.03 ± 1.28	28.38 ± 2.06	30.98 ± 2.09	23.63 ± 1.86	53.25 ± 3.18
MET	8.93 ± 1.44⁎⁎	18.67 ± 3.19⁎⁎	13.83 ± 1.84⁎⁎	12.07 ± 1.02⁎⁎	27.98 ± 3.24⁎⁎
XEC1	14.97 ± 1.16	30.08 ± 2.06	24.97 ± 2.04⁎⁎	20.27 ± 2.02*	47.64 ± 3.39*
XEC2	13.07 ± 1.71⁎⁎	27.28 ± 1.79	19.85 ± 2.03⁎⁎	15.62 ± 1.38⁎⁎	39.60 ± 3.34⁎⁎
XEC3	15.45 ± 1.50	28.45 ± 1.38	23.87 ± 1.96⁎⁎	17.78 ± 1.55⁎⁎	44.88 ± 3.03⁎⁎
XEC4	16.85 ± 2.23	31.50 ± 0.99	28.33 ± 1.40	24.68 ± 1.49	53.55 ± 1.74
XEC5	15.88 ± 0.91	30.15 ± 1.66	25.17 ± 1.81⁎⁎	19.32 ± 1.56⁎⁎	47.58 ± 2.91⁎⁎
XEC6	9.43 ± 1.67⁎⁎	19.58 ± 1.88⁎⁎	14.60 ± 0.88⁎⁎	11.82 ± 0.76⁎⁎	29.01 ± 2.24⁎⁎
XEC7	13.05 ± 1.47⁎⁎	27.65 ± 1.21	22.33 ± 1.28⁎⁎	17.83 ± 1.40⁎⁎	42.75 ± 2.53⁎⁎
XEC8	17.57 ± 3.87	31.80 ± 1.75	28.42 ± 2.24	23.58 ± 2.95	53.40 ± 4.74
XEC9	22.48 ± 1.80	32.56 ± 1.49	32.82 ± 0.83	30.57 ± 1.81	61.83 ± 2.05

The data were presented as means ± SD. T-test was applied in the assessment of the differences among multiple groups.

⁎⁎ *p*<0.01,

⁎ 0.01≤*p*<0.05, versus DC.

**Table 5:** Effects of different XEC formulae on HbA1C, TC and TG (*n* = 6).

Group	HbA1c (%)	TC (mmol/L)	TG (mmol/L)
NC	3.40 ± 0.23⁎⁎	2.64 ± 0.13⁎⁎	1.13 ± 0.19⁎⁎
DC	7.40 ± 1.20	7.18 ± 0.67	2.21 ± 0.19
MET	3.95 ± 0.67⁎⁎	4.47 ± 0.44⁎⁎	1.46 ± 0.07⁎⁎
XEC1	6.62 ± 0.84	5.68 ± 0.80⁎⁎	1.72 ± 0.10⁎⁎
XEC2	5.50 ± 0.94*	5.27 ± 0.83⁎⁎	1.67 ± 0.16⁎⁎
XEC3	6.23 ± 0.71	5.55 ± 1.00⁎⁎	1.75 ± 0.14⁎⁎
XEC4	7.43 ± 0.87	6.81 ± 0.86	1.98 ± 0.27⁎⁎
XEC5	6.62 ± 0.66	6.69 ± 0.85	1.60 ± 0.24⁎⁎
XEC6	4.03 ± 0.53⁎⁎	4.15 ± 0.57⁎⁎	1.41 ± 0.12⁎⁎
XEC7	5.93 ± 0.81*	5.29 ± 0.85⁎⁎	1.51 ± 0.12⁎⁎
XEC8	7.42 ± 0.80	5.87 ± 1.13*	1.82 ± 0.30*
XEC9	9.03 ± 0.86	6.75 ± 0.93	1.81 ± 0.24*

The data were presented as means ± SD. T-test was applied in the assessment of the differences among multiple groups.

⁎⁎ *p*<0.01,

⁎ 0.01≤*p*<0.05, versus DC.

Both the daily dose of each herb extract, or variables (X), and the metabolic parameters, or dependent variables (Y) were converted into normalized data before regression, eliminating the difference of units. Normalized data were obtained as followed:

$$A^{\prime }=\frac{A-A{\;}_{\text{min}}}{A_{\text{max}}\;-A_{\text{min}}}$$

The regression of normalized data was done using the software named as Data Processing System (DPS Version 7.05, Refine Information Tech. Co., China) [2]. Regression was done with the multivariate second degree polynomial model:

$$Y=a_{0}+\sum b_{i}x_{i}+\sum c_{i}x_{i}^{2}+\sum d_{\text{ij}}x_{i}x_{j}$$

Variables X_1_-X_4_, represent the normalized data of daily dose of four herb extracts. Y, or dependent variable, represents the normalized data of metabolic parameters. The extremum value of each regression model was calculated using grid algorithm with MATLAB 14.0.

Tables 6–10 presented statistic parameters of different regression equations concerning change of FBG, area under curve (AUC) of 2 h OGTT, levels of HbA1C, TC and TG. R value, R_a_ (adjusted R) value, p value and F value were calculated using DPS 7.0. For each metabolic parameter, an equation was selected as regression model for further analysis, based on a combined consideration on the R, R_a_, p and F value.

**Table 6:** Statistic parameters of different equations concerning change of FBG.

Equation\Parameters	*F*	R	R_a_	p
1-1	24.94	0.9287	0.9073	0.0010
1-2	21.70	0.9724	0.9497	0.0023
1-3	38.00	0.9896	0.9765	0.0018
1-4	34.53	0.9928	0.9784	0.0073
1-5	21.82	0.9935	0.9705	0.0445

Table 11 presented extremum values of selected equations and the corresponding optimal levels of four herb extracts. Table 12 presents predicted values of metabolic parameters using optimized daily dose of four herb extracts, and made a comparison to extremum values of selected equations.

Table 13 presented experimental results in the validation experiment. Raw data relating to validation experiment could be seen in Supplementary Materials, Tables S5-S7.

## 2 Experimental design, materials, and methods

### 2.1 Reagent and materials

Plant materials were obtained as our co-submitted article [1]. Four herbal drugs are listed as followed, i.e., Coptidis Rhizoma (*Huanglian*, dried rhizomes of *Coptis chinensis* Franch.), Liriopes Radix (*Maimendong*, dried roots of *Liriope spicata* (Thunb.) Lour. var. *prolifera*), bitter melon (*Kugua*, unripe fruits of *Momordica charantia* L.), and Cassiae Semen (*Juemingzi*, dried seeds of *Cassia obtusifolia* L.). Preparation of herb extracts reported below was in accordance to our previous report [3]. The four herb extracts were total alkaloids of Coptidis Rhizoma (TACR), Liriopes Radix polysaccharides (LRP), bitter melon extract (BME), and Cassiae Semen extract (CSE), respectively.

Streptozotocin (STZ) was purchased from Sigma-Aldrich Co. (St. Louis, MO, USA). Metformin was purchased from Bristol-Myers Squibb Co. (Shanghai, China). Rodent diet (D12451), a high-sucrose-high-fat (HSHF) diet, was obtained from Shulaibao Co. (Wuhan, China), with following formula: 23.306% casein, 0.350% cystine, 8.483% corn starch, 11.653% maltodextrin, 20.136% sucrose, 5.826% cellulose, 2.913% soybean oil, 20.684% lard, 1.165% multi-mine M1002, 1.515% calcium hydrogen phosphate, 0.641% calcium phosphate, 1.923% potassium citrate, 1.165% multi-vitamin, 0.233% choline tartrate and 0.005% edible blue dye. Energy ratio of proteins, carbohydrates and fats were 20 kcal%, 35 kcal% and 45 kcal%, respectively.

TC and TG kits were purchased from Nanjing Jiancheng Science and Technology Co. (Nanjing, China).

### 2.2 Animal and the establishment of diabetic model

Male Kunming mice (18–20 g) were purchased from Hubei Provincial Center for Disease Control and Prevention (No.42000600026992). The whole process of animal experiments was conducted in Laboratory Animal Center, Tongji Medical College, HUST (SYXK (Hubei) 2016-0057). Mice were acclimatized for seven days.

After acclimatization, eight mice, being normal mice, continued to receive the standard chow diet, while others received the HSHF diet. Two weeks later, HSHF-feeding mice were kept fast for 12 h and intraperitoneally (i.p.) injected STZ (120 mg/kg, pH 4.5, in citrate buffer). Normal mice were kept fast for 12 h, and subjected to injection of citrate buffer (i.p.). One week later, the level of FBG was measured via plasma glucose test strips (Bayer, Germany), with tail-tip blood. Then, mice with FBG below 11.1 mmol/L were subjected to i.p. STZ injection (40 mg/kg) again. One week later, mice with FBG above 11.1 mmol/L were regarded as diabetic.

### 2.3 Experimental design based on uniform design

#### 2.3.1 Selection of daily dose of herb extracts

In Chinese Pharmacopeia, the maximum dose of Coptidis Rhizoma (crude drug) is 5 g/d for human [4]. However, a clinical report pointed out that in clinical practice, clinicians choose a dose of 3–6 g/d in long term treatment of diabetes [5]. In this experiment, we chose 6 g/d as the maximum level of Coptidis Rhizoma (crude drug).

Cassiae Semen and Liriopes Radix have the same maximum daily dose for human, namely, 15 g/d according to Chinese Pharmacopeia [4].

Bitter melon is not recorded in 2015 Chinese Pharmacopeia, but recorded in 2018 Hubei Provincial Quality Standards of Chinese Materia Medica, with a maximum dose at 60 g/d for human [6].

Thus, maximum dose of each herb (crude drug, for human) was set as following: Coptidis Rhizoma, 6 g/d; Liriopes Radix and Cassiae Semen, 15 g/d; fresh bitter melon, 60 g/d. The dose of each crude drug for mouse was calculated according to the following equation:

$$D_{\text{mouse}}=D_{\text{human}}/70\text{kg}\times 9.1.$$

In this equation, D_mouse_ represents the dose of crude drug for mouse; D_human_ represents the dose of crude drug for human. Average human body weight is 70 kg. The dose conversion ratio of human to mice is 9.1 [7].

Considering the yield of each herb extracts reported in our previous research, the maximum dose of each herb extracts for mouse was set as followed: TACR, 136 mg/kg/d; LRP, 620 mg/kg/d; BME, 139 mg/kg/d; CSE, 72 mg/kg/d [1]. For ease of calculation, the minimum dose of each herb extracts was set at 1/10 of the maximum dose with small modification. Thus, the dose range of each herb extract was finally determined as followed: TACR, 16–136 mg/kg/d; LRP, 60–620 mg/kg/d; BME, 11–139 mg/kg/d; CSE, 8–72 mg/kg/d.

#### 2.3.2 Formula design based on uniform design

Twelve groups were included in this study, each group comprising of eight mice. Normal control group was abbreviated as NC, whereas diabetic control, DC. Mice in both groups were intragastrically given 0.1% water solution of sodium carboxymethyl cellulose (sodium CMC). Metformin were given at a dose of 150 mg/kg/d, with this group abbreviated as MET.

Nine XEC formulae were designed according to the principles of uniform design, abbreviated as XEC1-XEC9. Here, four variables, X_1_–X_4_, represent the daily dose of TACR, LRP, BME and CSE (mg/kg/d), respectively.

The scheme of uniform design U_9_(9^4^) was presented in Table 1. Combined with the U_9_(9^4^) scheme, daily dose of herb extracts was set accordingly in Table 2, with the ranges of daily dose of four herb extracts discussed in Section 2.3.2.

In uniform experimental design, sampling points had a uniform distribution within the range of each factor [8]. In other words, according to the principles of uniform experimental design used in this study, U_9_(9^4^), 9 levels distributed uniformly in the range of daily dose of herb extract. For example, the range of daily dose of TACR for mouse is 16–136 mg/kg/d. Therefore, level 1 of TACR is 16 mg/kg/d; level 2 of TACR is 31 mg/kg/d;…; while level 9 of TACR is 136 mg/kg/d. Nine levels distributed uniformly in the range. Combined with uniform experimental design table U_9_(9^4^) and the range of each herb extract, the daily dose of each herb extract was set and presented in Table 2. XEC1-XEC9 were prepared by mixing four herb extract in a ratio in accordance to Table 2, and then were suspended in 0.1% sodium CMC before intragastrical administration to mice.

During the experimental process, solution was intragastrically given at 10 ml/kg/d, once a day. At the end of the study, blood was collected from the retro-orbital sinus, and mice were sacrificed thereafter. A part of blood sample was centrifugated for serum (4500 rpm, 10 min), while another part was placed in tubes containing EDTA, as whole blood.

Animal experiments were conducted under the guidance of Regulations for the Administration of Affairs Concerning Experimental Animals in Hubei Province. Experimental procedures were carried out with approval from the Institutional Animal Care and Use Committee, Huazhong University of Science and Technology.

### 2.4 Measurement of FBG and performance of OGTT

FBG was monitored every week after fasting for 12 h. Raw data relating to FBG could be seen in Supplementary Materials, Table S1. Levels of FBG in day 0 were different. Thus, regression was done with change of FBG, instead of FBG, eliminating the differences in FBG in day 0. Change of FBG was calculated as:

$$Y=\frac{\text{FB}G_{0d}-\text{FB}G_{28d}\;}{\text{FB}G_{0d}}\times \;100\%$$

OGTT was carried out on mice fasting for 12 h overnight, on the 24th day. Raw data relating to OGTT could be seen in Supplementary Materials, Table S2. Mice were intragastrically administered with glucose solution (2.5 g/kg). Blood glucose (BG) was monitored at three time points: 0.5, 1 and 2 h, after glucose administration. BG levels before glucose administration were regarded as 0 h. Calculation of area under curve (AUC) was conducted as followed:

$$\text{AUC}=0.25\;\times BG_{0\;h}+0.5\;\times BG_{0.5\;h}+0.75\;\times BG_{1\;h}+0.5\;\times BG_{2\;h}$$

### 2.5 Measurement of HbA1c, TC and TG

HbA1c measurement was conducted with whole blood, via Ultra2 GHb meter (Primus, USA). TC and TG in serum were analyzed using commercial kits from Nanjing Jiancheng Science and Technology Co. (Nanjing, China). Raw data relating to these parameters could be seen in Supplementary Materials, Tables S3 and S4.

### 2.6 Fitting of model and regression

#### 2.6.1 Fitting and regression on the changes of FBG

As can be seen from Table 3, mice in each group had different FBG levels in day 0. Thus, changes of FBG were applied in the regression, eliminating the influence of different FBG levels in day 0. When the change of FBG is positive, it shows a hypoglycemic effect, if negative, hyperglycemic effect.

Y represents the changes of FBG, while X_1_-X_4_ represent the daily dose of four herb extracts. All variables were normalized and regression was done with DPS 7.0. Several multivariate second degree polynomial functions were obtained.

R, R_a_, p and F value were also reported in Table 6. Equation (1-4) was selected as regression model of change of FBG for further analysis.

(1-1) $$Y=0.580+1.228\;X_{2}-1{.788\;X}_{2}^{2}$$

(1-2) $$Y=0.502+0.776\;X_{1}+0.957\;X_{2}-0{.708\;X}_{1}^{2}-1{.547\;X}_{2}^{2}$$

(1-3) $$Y=0.478+0.854\;X_{1}+1.141\;X_{2}-0{.940\;X}_{1}^{2}-1{.866\;X}_{2}^{2}+0.346\;X_{1}\times X_{3}$$

(1-4) $$Y=0.489+0.823\;X_{1}+1.255\;X_{2}-0{.999\;X}_{1}^{2}-1{.972\;X}_{2}^{2}-0{.126\;X}_{3}^{2}+0.535\;X_{1}\times X_{3}$$

(1-5) $$Y=0.508+1.504\;X_{1}+0.988\;X_{2}-2{.470\;X}_{1}^{2}-2{.372\;X}_{2}^{2}-0{.110\;X}_{3}^{2}+1.426\;X_{1}\times X_{2}+0.529\;X_{1}\times X_{3}$$

#### 2.6.2 Fitting and regression on 2h OGTT AUC

Y_AUC_ represents the AUC of 2 h OGTT and X_1_-X_4_ represent the daily dose of four herb extracts. Regression was done as previous mentioned. R, R_a_, p and F value were also reported in Table 7. The equation (2-1) was selected for further analysis.

(2-1) $$Y_{\text{AUC}}=0.753-1.004\;X_{1}-1.659\;X_{2}+1{.256\;X}_{1}^{2}+2{.478\;X}_{2}^{2}-0.796\;X_{1}\times X_{3}$$

(2-2) $$Y_{\text{AUC}}=0.769-0.422\;X_{1}-1.874\;X_{2}+2{.133\;X}_{2}^{2}+1.198\;X_{1}\times X_{2}-0.778\;X_{1}\times X_{3}$$

(2-3) $$Y_{\text{AUC}}=0.691+2.044\;X_{2}-0{.999\;X}_{1}^{2}+2{.548\;X}_{2}^{2}+0.629\;X_{1}\times X_{2}-0.835\;X_{1}\times X_{3}$$

(2-4) $$Y_{\text{AUC}}=0.767-1.147\;X_{1}-1.620\;X_{2}+1{.418\;X}_{1}^{2}+2{.443\;X}_{2}^{2}-0.690\;X_{1}\times X_{3}-0.170\;X_{2}\times X_{4}$$

**Table 7:** Statistic parameters of different equations concerning 2h OGTT AUC.

Equation\Parameters	F	R	R_a_	p
2-1	20.75	0.9813	0.9573	0.0058
2-2	19.37	0.9800	0.9543	0.0066
2-3	10.76	0.9465	0.9014	0.0113
2-4	16.64	0.9853	0.9553	0.0210

#### 2.6.3 Fitting and regression on HbA1c

Y represents the HbA1c level and X_1_-X_4_ represents the daily dose of four herb extracts. Regression was done as previous mentioned. R, R_a_, p and F value were also reported in Table 8. The equation (3-3) was selected for further analysis.

(3-1) $$Y_{A1c}=0.606-1.745\;X_{2}+2{.467\;X}_{2}^{2}-0.379\;X_{1}\times X_{3}$$

(3-2) $$Y_{A1c}=0.632-1.942\;X_{2}+2{.398\;X}_{2}^{2}+0.602\;X_{1}\times X_{2}-0.716\;X_{1}\times X_{3}$$

(3-3) $$Y_{A1c}=0.694-0.982\;X_{1}-1.565\;X_{2}+1{.222\;X}_{1}^{2}+2{.323\;X}_{2}^{2}-0.678\;X_{1}\times X_{3}$$

(3-4) $$Y_{A1c}=0.709-0.416\;X_{1}-1.775\;X_{2}+1{.989\;X}_{2}^{2}+1.162\;X_{1}\times X_{2}-0.660\;X_{1}\times X_{3}$$

(3-5) $$Y_{A1c}=0.700-1.048\;X_{1}-1.548\;X_{2}+1{.297\;X}_{1}^{2}+2{.307\;X}_{2}^{2}-0.630\;X_{1}\times X_{3}-0.078\;X_{2}\times X_{4}$$

**Table 8:** Statistic parameters of different equations concerning HbA1c.

Equation\Parameters	F	R	R_a_	p
3-1	9.76	0.9110	0.8630	0.0101
3-2	11.71	0.9505	0.9090	0.0094
3-3	28.57	0.9863	0.9689	0.0032
3-4	25.54	0.9847	0.9652	0.0039
3-5	19.14	0.9872	0.9611	0.0172

#### 2.6.4 Fitting and regression on TC

Y represents the TC level and X_1_-X_4_ represent the daily dose of four herb extracts. Regression was done as previous mentioned. R, R_a_, p and F value were also reported in Table 9. The equation (4-4) was selected for further analysis.

(4-1) $$Y_{\text{TC}}=0.897-2.684\;X_{2}+3{.487\;X}_{2}^{2}-0.863\;X_{1}\times X_{3}$$

(4-2) $$Y_{\text{TC}}=0.918-2.837\;X_{2}+3{.434\;X}_{2}^{2}+0.466\;X_{1}\times X_{2}-1.124\;X_{1}\times X_{3}$$

(4-3) $$Y_{\text{TC}}=0.919-3.002\;X_{2}+3{.485\;X}_{2}^{2}+0{.204\;X}_{3}^{2}+0.690\;X_{1}\times X_{2}-1.419\;X_{1}\times X_{3}$$

(4-4) $$Y_{\text{TC}}=0.990-3.098\;X_{2}-0.142\;X_{4}+3{.435\;X}_{2}^{2}+0{.256\;X}_{3}^{2}+0.755\;X_{1}\times X_{2}-1.346\;X_{1}\times X_{3}$$

**Table 9:** Statistic parameters of different equations concerning TC.

Equation\Parameters	F	R	R_a_	p
4-1	20.48	0.9545	0.9309	0.0015
4-2	22.12	0.9729	0.9506	0.0022
4-3	20.07	0.9806	0.9574	0.0062
4-4	21.94	0.9888	0.9660	0.0142

#### 2.6.5 Fitting and regression on TG

Y represents the TG level and X_1_-X_4_ represent the daily dose of four herb extracts. Regression was done as previous mentioned. R, R_a_, p and F value were also reported in Table 10. The equation (5-2) was selected for further analysis.

(5-1) $$Y_{\text{TG}}=0.937-0.335\;X_{1}-2.392\;X_{4}+1{.593\;X}_{4}^{2}$$

(5-2) $$Y_{\text{TG}}=0.996-0.998\;X_{1}-2.061\;X_{4}+0{.603\;X}_{1}^{2}+1{.593\;X}_{4}^{2}$$

(5-3) $$Y_{\text{TG}}=0.958-0.506\;X_{1}-2.185\;X_{4}+1{.694\;X}_{4}^{2}+0.532\;X_{2}\times X_{4}$$

(5-4) $$Y_{\text{TG}}=0.958-1.348\;X_{1}-1.793\;X_{3}+1{.007\;X}_{1}^{2}+0{.173\;X}_{2}^{2}+\;1{.464\;X}_{4}^{2}$$

(5-5) $$Y_{\text{TG}}=1.010-1.211\;X_{1}-0.251\;X_{3}-1.959\;X_{4}+0{.936\;X}_{1}^{2}+0{.373\;X}_{3}^{2}+\;1{.566\;X}_{4}^{2}$$

**Table 10:** Statistic parameters of different equations concerning TG.

Equation\Parameters	F	R	R_a_	p
5-1	12.75	0.9297	0.8925	0.0052
5-2	3686.22	0.9999	0.9998	0.0001
5-3	59.11	0.9896	0.9812	0.0002
5-4	46.86	0.9916	0.9809	0.0012
5-5	35.33	0.9930	0.9788	0.0071

### 2.7 Extremum value of selected equation and the corresponding optimized level of four herb extracts

Extremum values of selected equations and the corresponding values of four herb extracts were calculated using grid algorithm in MATLAB 14.0. Results were shown in Table 11. For change of FBG, when the value is positive, it shows a hypoglycemic effect. In other words, a higher value means a better glycemic control. Thus, the maximum value of change of FBG was calculated. For other four parameters, the minimum values were calculated. Herein, the optimal daily dose of each herb extract was set with consideration on experimental data, regression results and calculation of extremum values. The optimized daily dose of XEC for mouse was set as following: TACR, 95.5 mg/kg/d; LRP, 244.8 mg/kg/d; BME, 139.0 mg/kg/d; CSE, 49.1 mg/kg/d (Table 12). Thus, optimized formula of XEC was set accordingly, TACR: LRP: BME: CSE 0.181: 0.463: 0.263: 0.093. In addition, predicted values of metabolic parameters using optimized formula were also calculated in Table 12.

**Table 11:** Extremum value of each equation and the corresponding daily dose of four herb extracts.

Parametes	Extremum	Extremum (standardized)	Corresponding daily dose (mg/kg/d)				Corresponding daily dose (standarized)			
			TACR	LRP	BME	CSE	TACR	LRP	BME	CSE
Change of FBG (%)	42.89	1.0244	98.0	238.1	139.0	–	0.6834	0.3180	1.0000	-
2h OGTT AUC (mmol/L•h)	23.46	−0.1690	101.5	247.6	139.0	–	0.7127	0.3350	1.0000	-
HbA1c (%)	3.36	−0.1332	100.0	254.8	139.0	–	0.6999	0.3479	1.0000	-
TC (mmol/L)	2.21	−0.6409	136.0	251.9	139.0	72.0	1.0000	0.3426	1.0000	1.0000
TG (mmol/L)	1.05	−0.0831	116.4	–	–	49.1	0.8363	–	–	0.6424

Note: “-” represent the absence of the factor.

**Table 12:** Predicted values of selected equations in optimized doses of herb extracts.

	Herb extracts				Parameters	Selected equation	Extremum of selected equations	Predicted value using optimized dose
	TACR	LRP	BME	CSE				
Optimized dose in standardized data	0.6625	0.3300	1.000	0.6424	Change of FBG (%)	1-4	42.89	42.83
					2h OGTT AUC (mmol/L•h)	2-1	23.46	23.55
Optimized dose (mg/kg/d)	95.5	244.8	139.0	49.1	HbA1c (%)	3-3	3.36	3.37
					TC (mmol/L)	4-4	2.21	2.45
Ratio	0.181	0.463	0.263	0.093	TG (mmol/L)	5-2	1.05	1.36

**Table 13:** Effects of XEC (V) on diabetic mice in the validation experiment.

Group\Parameters	FBG (mmol/L)		Change of FBG (%)	2h OGTT AUC (mmol/L•h)	HbA1c (%)	TC (mmol/L)	TG (mmol/L)
	Day 0	Day 28					
NCV	3.73 ± 0.31⁎⁎	3.79 ± 0.27⁎⁎	–	13.87 ± 0.83⁎⁎	3.23 ± 0.23⁎⁎	2.62 ± 0.21⁎⁎	1.08 ± 0.14⁎⁎
DCV	16.45 ± 1.87	16.75 ± 1.34	−1.82	51.52 ± 3.58	7.31 ± 0.83	7.20 ± 0.83	2.34 ± 0.17
METV	16.15 ± 1.74⁎⁎	9.30 ± 1.73⁎⁎	42.41	27.34 ± 2.76⁎⁎	3.93 ± 0.29⁎⁎	4.09 ± 0.50⁎⁎	1.50 ± 0.10⁎⁎
XECV	15.86 ± 1.82⁎⁎	8.76 ± 1.45⁎⁎	44.77	24.93 ± 3.09⁎⁎	3.58 ± 0.35⁎⁎	2.73 ± 0.43⁎⁎^,^##	1.35 ± 0.08⁎⁎
Predicted value	–	9.06	42.83	23.55	3.37	2.45	1.36
Deviation between predicted value and experimental data (%)	–	−3.31	4.53	5.86	6.23	11.4	−0.74

The data were presented as means ± SD. T-test was applied in the assessment of the differences among multiple groups.

⁎⁎ *p*<0.01, *0.01≤*p*<0.05, versus DCV.

## *p*<0.01, ^#^0.01≤*p*<0.05, versus METV.

### 2.8 Validation experiment

An extract combination was prepared by mixing four herb extracts in a ratio of TACR: LRP: BME: CSE 0.181: 0.463: 0.263: 0.093. XEC were suspended in 0.1% sodium CMC. According to the optimized daily dose of four herb extracts, XEC was given at a dose of 528 mg/kg/d.

After establishment of diabetic model as previously mentioned, 32 male Kunming mice were categorized into 4 groups, i.e., normal control for validation (NCV), diabetic control for validation (DCV), metformin (150 mg/kg/d) for validation (METV) and XEC (528 mg/kg/d) for validation (XECV). Levels of blood glucose, HbA1c, TC, TG were measured as previously mentioned. OGTT was performed as previously mentioned. Raw data relating to these parameters could be seen in Supplementary Materials, Tables S5–S7.

Predicted value in XECV was calculated using regression equations selected above. Deviation between predicted value and experimental data (%) was calculated as followed:

Deviation(%) = (Predicted value- Experimental data)/ Predicted value

Results of the validation experiment were shown in Table 13.

## Ethics statement

Animal experiments were conducted under the guidance of Regulations for the Administration of Affairs Concerning Experimental Animals in Hubei Province. Experimental procedures were carried out with approval from the Institutional Animal Care and Use Committee, Huazhong University of Science and Technology, with IACUC number: 831.

## Declaration of Competing Interest

The authors declare that they have no known competing financial interests or personal relationships which have, or could be perceived to have, influenced the work reported in this article.

## Appendix Supplementary materials

Image, application 1
