# Supplementary material for: Data on the optimization of the formula of Xiaokeyinshui extract combination treating diabetes mellitus using uniform experimental design in mice
Source: Data Brief. 2020 Aug 5;32:106134. doi: 10.1016/j.dib.2020.106134 (PMC7452698; doi:10.1016/j.dib.2020.106134)
Supplement: Supplementary file 1 [file mmc1.docx]

**Appendix A. Supplementary Materials**

**Data on the optimization of the formula of *Xiaokeyinshui* extract combination treating diabetes mellitus using uniform experimental design in mice**

**Authors**

Jiewen Zhou, Jun Pan, Zhinan Xiang, Qiuyan Wang, Qilin Tong, Jinbo Fang, Luosheng Wan*, Jiachun, Chen*

**Affiliations**

Hubei Key Laboratory of Natural Medicinal Chemistry and Resource Evaluation, College of Pharmacy, Huazhong University of Science and Technology, Hangkong Road 13, Wuhan 430030, Hubei Province, China.

**Corresponding authors**

Correspondence should be addressed to Jiachun Chen (homespringchen@mail.hust.edu.cn) and Luosheng Wan (wanlesheng1@163.com).

**Table S1. Raw data of fasting blood glucose (FBG)**

**in the experiment of uniform design**

| Group | Mice No. | FBG (mmol/L) | | | | |
| --- | --- | --- | --- | --- | --- | --- |
|  |  | Day 0 | Day 7 | Day 14 | Day 21 | Day 28 |
| NC | A001 | 3.7 | 4.1 | 3.8 | 2.6 | 4.6 |
|  | A002 | 4.2 | 3.9 | 3.1 | 3.5 | 3.2 |
|  | A003 | 3.1 | 3.8 | 4.2 | 2.4 | 3.6 |
|  | A004 | 4.5 | 4.8 | 2.8 | 3.1 | 3.1 |
|  | A005 | 3.6 | 4.1 | 2.9 | 2.9 | 3.9 |
|  | A006 | 4.2 | 3.4 | 3.0 | 3.2 | 3.4 |
|  | A007 | 3.3 | 3.7 | 2.8 | 4.0 | 3.7 |
|  | A008 | 3.7 | 3.7 | 3.0 | 3.3 | 3.2 |
| DC | A012 | 14.2 | 14.2 | 13.0 | 11.8 | 13.9 |
|  | A051 | 13.3 | 14.0 | 14.2 | 12.7 | 14.6 |
|  | A054 | 14.7 | 16.9 | 16.8 | 17.0 | 15.2 |
|  | A082 | 16.1 | 15.3 | 16.4 | 16.8 | 15.8 |
|  | A090 | 17.0 | 18.9 | 18.7 | 18.7 | 17.2 |
|  | A099 | 17.4 | 18.0 | 18.4 | 19.9 | 17.5 |
|  | A102 | 15.6 | 16.4 | 14.4 | 12.7 | 16.0 |
|  | A112 | 15.9 | 16.1 | 15.3 | 16.0 | 15.8 |
| MET | A046 | 15.7 | 12.4 | 11.6 | 10.5 | 10.4 |
|  | A058 | 12.9 | 12.2 | 9.9 | 9.3 | 6.7 |
|  | A070 | 16.6 | 13.3 | 12.5 | 11.2 | 7.0 |
|  | A071 | 16.9 | 14.9 | 11.6 | 11.1 | 11.8 |
|  | A079 | 19.0 | 15.6 | 13.0 | 9.8 | 6.1 |
|  | A132 | 11.8 | 11.6 | 9.5 | 8.2 | 10.9 |
|  | A136 | 16.7 | 13.3 | 11.0 | 10.2 | 8.8 |
|  | A150 | 14.2 | 14.4 | 11.4 | 10.5 | 7.8 |
| XEC1 | A015 | 16.4 | 17.2 | 18.2 | 17.4 | 15.2 |
|  | A031 | 14.3 | 16.2 | 16.9 | 16.5 | 15.0 |
|  | A059 | 15.5 | 16.4 | 16.9 | 14.2 | 13.3 |
|  | A067 | 12.3 | 13.1 | 12.1 | 12.8 | 11.2 |
|  | A080 | 18.2 | 19.5 | 19.3 | 16.8 | 17.8 |
|  | A103 | 14.1 | 15.6 | 14.4 | 13.4 | 12.9 |
|  | A108 | 13.2 | 15.1 | 12.1 | 12.0 | 11.0 |
|  | A127 | 16.1 | 15.9 | 15.6 | 15.4 | 14.7 |
| XEC2 | A013 | 14.5 | 15.3 | 11.5 | 9.2 | 11.2 |
|  | A016 | 14.9 | 16.6 | 13.8 | 13.5 | 13.3 |
|  | A032 | 15.2 | 16.3 | 14.6 | 14.7 | 14.6 |
|  | A072 | 17.2 | 18.1 | 19.3 | 15.7 | 16.5 |
|  | A128 | 14.8 | 17.0 | 14.4 | 14.6 | 12.0 |
|  | A133 | 16.0 | 16.2 | 13.3 | 14.2 | 11.2 |
|  | A141 | 18.6 | 18.2 | 18.5 | 17.5 | 16.0 |
|  | A157 | 13.5 | 14.4 | 12.7 | 13.1 | 10.8 |
| XEC3 | A035 | 17.2 | 16.9 | 16.6 | 15.3 | 14.1 |
|  | A039 | 19.3 | 22.2 | 20.2 | 19.7 | 18.1 |
|  | A043 | 13.3 | 16.8 | 15.8 | 10.0 | 12.4 |
|  | A047 | 15.7 | 19.2 | 17.7 | 16.5 | 15.5 |
|  | A085 | 11.9 | 13.8 | 14.9 | 13.8 | 13.6 |
|  | A088 | 17.3 | 19.3 | 16.9 | 16.5 | 14.7 |
|  | A149 | 20.4 | 19.6 | 18.7 | 18.3 | 15.7 |
|  | A156 | 16.5 | 19.0 | 16.7 | 16.6 | 15.1 |
| XEC4 | A019 | 16.3 | 18.4 | 18.2 | 16.8 | 18.9 |
|  | A026 | 12.2 | 16.1 | 14.4 | 11.7 | 14.7 |
|  | A029 | 21.0 | 20.1 | 21.7 | 21.9 | 22.8 |
|  | A030 | 13.5 | 18.4 | 16.5 | 16.2 | 14.9 |
|  | A055 | 17.1 | 19.8 | 20.8 | 20.8 | 19.3 |
|  | A106 | 16.6 | 19.2 | 20.2 | 20.4 | 19.0 |
|  | A118 | 16.2 | 19.7 | 18.6 | 18.4 | 18.3 |
|  | A148 | 12.4 | 18.0 | 15.1 | 15.3 | 14.9 |
| XEC5 | A027 | 19.8 | 20.8 | 20.9 | 20.2 | 17.5 |
|  | A050 | 13.0 | 14.3 | 11.5 | 14.4 | 14.1 |
|  | A109 | 17.1 | 17.4 | 17.2 | 17.0 | 16.0 |
|  | A122 | 18.0 | 18.5 | 15.7 | 16.7 | 16.3 |
|  | A129 | 18.5 | 17.4 | 17.6 | 15.9 | 15.4 |
|  | A139 | 22.5 | 20.0 | 19.3 | 17.5 | 17.4 |
|  | A140 | 14.9 | 14.7 | 15.5 | 15.4 | 14.8 |
|  | A155 | 17.3 | 20.8 | 17.7 | 15.7 | 15.4 |
| XEC6 | A034 | 14.7 | 14.5 | 10.3 | 11.4 | 8.4 |
|  | A041 | 16.7 | 15.5 | 11.7 | 11.5 | 10.7 |
|  | A060 | 13.5 | 14.2 | 9.9 | 11.0 | 7.0 |
|  | A092 | 15.4 | 14.8 | 11.4 | 12.8 | 10.3 |
|  | A107 | 18.0 | 16.9 | 15.3 | 14.8 | 11.2 |
|  | A111 | 15.2 | 15.6 | 14.6 | 12.7 | 8.6 |
|  | A142 | 19.5 | 18.2 | 15.9 | 13.6 | 10.7 |
|  | A158 | 17.8 | 16.8 | 14.9 | 12.0 | 10.5 |
| XEC7 | A040 | 14.6 | 16.2 | 12.4 | 13.1 | 11.6 |
|  | A053 | 16.3 | 18.1 | 16.1 | 15.3 | 14.3 |
|  | A083 | 13.3 | 14.2 | 12.6 | 13.2 | 11.2 |
|  | A097 | 15.0 | 17.7 | 16.6 | 15.1 | 14.3 |
|  | A100 | 18.7 | 18.4 | 17.5 | 16.6 | 16.4 |
|  | A105 | 16.9 | 16.8 | 13.9 | 13.7 | 12.5 |
|  | A110 | 20.3 | 18.7 | 18.4 | 16.4 | 16.1 |
|  | A119 | 16.9 | 18.3 | 16.9 | 13.4 | 14.4 |
| XEC8 | A014 | 13.4 | 15.1 | 15.2 | 15.4 | 14.3 |
|  | A023 | 14.6 | 17.6 | 16.7 | 16.3 | 14.9 |
|  | A096 | 16.2 | 17.9 | 16.7 | 17.6 | 14.9 |
|  | A113 | 13.0 | 13.5 | 14.2 | 14.4 | 14.1 |
|  | A115 | 13.7 | 18.8 | 17.7 | 18.2 | 17.4 |
|  | A116 | 20.9 | 19.0 | 18.1 | 18.2 | 21.7 |
|  | A134 | 13.9 | 17.4 | 15.5 | 17.0 | 14.4 |
|  | A153 | 20.9 | 21.2 | 19.7 | 22.0 | 22.9 |
| XEC9 | A018 | 15.9 | 18.4 | 19.8 | 22.2 | 22.1 |
|  | A061 | 21.3 | 19.2 | 21.8 | 24.4 | 28.6 |
|  | A078 | 14.0 | 18.1 | 19.3 | 18.4 | 20.1 |
|  | A081 | 11.9 | 14.2 | 15.6 | 16.4 | 17.5 |
|  | A104 | 13.3 | 17.3 | 20.4 | 18.2 | 20.1 |
|  | A137 | 12.7 | 16.8 | 18.7 | 16.9 | 17.8 |
|  | A144 | 20.1 | 20.7 | 25.7 | 27.1 | 31.4 |
|  | A152 | 15.5 | 18.7 | 20.0 | 22.3 | 23.3 |

**Table S2. Raw data of oral glucose tolerance test (OGTT)**

**in the experiment of uniform design (n=6)**

| Group | Mice No. | Blood glucose (mmol/L) | | | |
| --- | --- | --- | --- | --- | --- |
|  |  | 0 h | 0.5 h | 1 h | 2 h |
| NC | A001 | 3.3 | 7.8 | 8.1 | 6.0 |
|  | A002 | 3.5 | 11.2 | 6.4 | 7.4 |
|  | A004 | 3.6 | 12.1 | 7.9 | 5.6 |
|  | A005 | 4.0 | 10.6 | 7.4 | 4.6 |
|  | A006 | 3.1 | 9.1 | 7.8 | 4.9 |
|  | A007 | 3.4 | 9.7 | 6.9 | 5.1 |
| DC | A054 | 15.5 | 25.3 | 28.0 | 20.6 |
|  | A082 | 15.0 | 30.2 | 32.6 | 22.4 |
|  | A090 | 17.2 | 30.4 | 32.9 | 23.8 |
|  | A099 | 17.9 | 27.2 | 31.3 | 25.6 |
|  | A102 | 16.0 | 29.8 | 32.3 | 25.1 |
|  | A112 | 14.6 | 27.4 | 28.8 | 24.3 |
| MET | A046 | 8.7 | 19.7 | 14.5 | 12.6 |
|  | A058 | 8.4 | 15.1 | 12.5 | 11.7 |
|  | A071 | 11.5 | 23.2 | 16.8 | 12.2 |
|  | A132 | 8.4 | 17.9 | 14.7 | 11.4 |
|  | A136 | 9.4 | 20.8 | 12.0 | 13.7 |
|  | A150 | 7.2 | 15.3 | 12.5 | 10.8 |
| XEC1 | A015 | 16.3 | 30.6 | 24.1 | 20.7 |
|  | A031 | 14.8 | 30.4 | 27.7 | 20.8 |
|  | A059 | 15.8 | 31.3 | 26.5 | 22.8 |
|  | A103 | 14.1 | 26.3 | 23.4 | 18.5 |
|  | A108 | 13.2 | 29.6 | 22.3 | 17.3 |
|  | A127 | 15.6 | 32.3 | 25.8 | 21.5 |
| XEC2 | A013 | 13.8 | 27.0 | 20.2 | 16.5 |
|  | A016 | 11.4 | 25.3 | 17.9 | 15.1 |
|  | A072 | 15.3 | 29.8 | 22.3 | 16.9 |
|  | A128 | 11.0 | 26.9 | 18.1 | 15.4 |
|  | A133 | 14.4 | 29.0 | 22.2 | 16.6 |
|  | A157 | 12.5 | 25.7 | 18.4 | 13.2 |
| XEC3 | A039 | 17.3 | 28.4 | 24.9 | 17.6 |
|  | A043 | 13.2 | 26.9 | 21.3 | 15.9 |
|  | A085 | 14.5 | 28.2 | 24.5 | 18.1 |
|  | A088 | 15.1 | 27.1 | 22.4 | 16.8 |
|  | A149 | 15.9 | 30.3 | 26.8 | 20.5 |
|  | A156 | 16.7 | 29.8 | 23.3 | 17.8 |
| XEC4 | A019 | 18.7 | 30.0 | 28.6 | 24.3 |
|  | A026 | 13.6 | 32.3 | 26.2 | 26.1 |
|  | A030 | 16.5 | 31.7 | 27.4 | 22.3 |
|  | A055 | 19.8 | 32.5 | 28.6 | 25.3 |
|  | A118 | 17.1 | 31.9 | 30.3 | 26.2 |
|  | A148 | 15.4 | 30.6 | 28.9 | 23.9 |
| XEC5 | A050 | 14.6 | 27.8 | 22.9 | 16.7 |
|  | A109 | 16.7 | 32.6 | 27.4 | 20.5 |
|  | A122 | 15.6 | 30.2 | 25.6 | 21.2 |
|  | A139 | 17.1 | 31.3 | 27.0 | 18.8 |
|  | A140 | 15.4 | 29.4 | 24.3 | 19.1 |
|  | A155 | 15.9 | 29.6 | 23.8 | 19.6 |
| XEC6 | A034 | 8.0 | 18.7 | 14.3 | 11.9 |
|  | A041 | 10.1 | 20.1 | 15.3 | 12.4 |
|  | A060 | 7.3 | 16.4 | 13.6 | 10.9 |
|  | A107 | 11.6 | 21.9 | 15.3 | 12.7 |
|  | A111 | 8.8 | 19.8 | 13.6 | 10.9 |
|  | A158 | 10.8 | 20.6 | 15.5 | 12.1 |
| XEC7 | A040 | 10.9 | 26.3 | 20.3 | 16.3 |
|  | A053 | 14.2 | 28.2 | 23.7 | 18.8 |
|  | A083 | 11.8 | 26.4 | 21.6 | 16.6 |
|  | A097 | 13.6 | 27.4 | 22.3 | 18.5 |
|  | A110 | 14.8 | 29.5 | 23.6 | 19.8 |
|  | A119 | 13.0 | 28.1 | 22.5 | 17.0 |
| XEC8 | A014 | 15.2 | 30.8 | 26.3 | 19.2 |
|  | A096 | 14.4 | 30.9 | 25.9 | 21.4 |
|  | A115 | 20.7 | 33.3 | 30.8 | 25.5 |
|  | A116 | 20.4 | 33.3 | 30.7 | 25.8 |
|  | A134 | 12.8 | 29.2 | 27.1 | 22.8 |
|  | A153 | 21.9 | 33.3 | 29.7 | 26.8 |
| XEC9 | A018 | 24.1 | 33.3 | 33.3 | 32.7 |
|  | A078 | 20.1 | 29.6 | 33.3 | 29.3 |
|  | A081 | 23.2 | 33.0 | 33.3 | 30.9 |
|  | A104 | 22.1 | 33.3 | 31.3 | 28.0 |
|  | A137 | 20.8 | 33.3 | 32.4 | 30.1 |
|  | A152 | 24.6 | 33.3 | 33.3 | 32.4 |

**Table S3. Raw data of HbA1c in the experiment of uniform design (n=6)**

| Group | Mice No. | HbA1c (%) |  | Group | Mice No. | HbA1c (%) |
| --- | --- | --- | --- | --- | --- | --- |
| NC | A002 | 3.0 |  | XEC4 | A026 | 6.7 |
|  | A003 | 3.4 |  |  | A029 | 7.8 |
|  | A004 | 3.3 |  |  | A030 | 6.8 |
|  | A005 | 3.5 |  |  | A055 | 9.0 |
|  | A006 | 3.6 |  |  | A118 | 7.4 |
|  | A007 | 3.6 |  |  | A148 | 6.9 |
| DC | A012 | 5.7 |  | XEC5 | A050 | 5.8 |
|  | A054 | 7.4 |  |  | A109 | 7.0 |
|  | A090 | 8.5 |  |  | A122 | 7.4 |
|  | A099 | 9.0 |  |  | A129 | 7.2 |
|  | A102 | 6.7 |  |  | A140 | 6.1 |
|  | A112 | 7.1 |  |  | A155 | 6.2 |
| MET | A046 | 5.0 |  | XEC6 | A034 | 3.6 |
|  | A058 | 3.3 |  |  | A041 | 4.2 |
|  | A070 | 3.4 |  |  | A060 | 3.3 |
|  | A079 | 3.7 |  |  | A111 | 4.0 |
|  | A132 | 4.5 |  |  | A142 | 4.8 |
|  | A136 | 3.8 |  |  | A158 | 4.3 |
| XEC1 | A015 | 7.4 |  | XEC7 | A040 | 5.6 |
|  | A031 | 7.3 |  |  | A053 | 5.7 |
|  | A059 | 6.9 |  |  | A083 | 5.0 |
|  | A067 | 6.4 |  |  | A097 | 7.4 |
|  | A103 | 6.6 |  |  | A105 | 5.7 |
|  | A108 | 5.1 |  |  | A119 | 6.2 |
| XEC2 | A013 | 4.9 |  | XEC8 | A014 | 6.9 |
|  | A016 | 5.2 |  |  | A096 | 7.3 |
|  | A032 | 5.9 |  |  | A113 | 6.6 |
|  | A128 | 5.9 |  |  | A115 | 7.5 |
|  | A141 | 6.9 |  |  | A134 | 7.3 |
|  | A157 | 4.2 |  |  | A153 | 8.9 |
| XEC3 | A043 | 5.0 |  | XEC9 | A018 | 9.7 |
|  | A047 | 6.8 |  |  | A078 | 9.6 |
|  | A085 | 6.0 |  |  | A081 | 8.2 |
|  | A088 | 6.3 |  |  | A104 | 8.8 |
|  | A149 | 7.0 |  |  | A137 | 7.9 |
|  | A156 | 6.3 |  |  | A152 | 10.0 |

**Table S4. Raw data of TC and TG in the experiment of uniform design (n=6)**

| Group | Mice No. | TC (mmol/L) | TG (mmol/L) |
| --- | --- | --- | --- |
| NC | A002 | 2.703 | 1.233 |
|  | A003 | 2.649 | 0.974 |
|  | A004 | 2.597 | 1.278 |
|  | A005 | 2.661 | 1.394 |
|  | A006 | 2.805 | 0.952 |
|  | A007 | 2.421 | 0.953 |
| DC | A012 | 6.281 | 2.153 |
|  | A051 | 6.452 | 1.927 |
|  | A054 | 7.463 | 2.226 |
|  | A082 | 7.479 | 2.233 |
|  | A090 | 7.408 | 2.177 |
|  | A099 | 8.012 | 2.529 |
| MET | A046 | 4.799 | 1.565 |
|  | A070 | 4.306 | 1.391 |
|  | A071 | 4.163 | 1.451 |
|  | A079 | 4.111 | 1.421 |
|  | A132 | 4.224 | 1.401 |
|  | A136 | 5.219 | 1.515 |
| XEC1 | A015 | 6.803 | 1.871 |
|  | A031 | 5.505 | 1.705 |
|  | A059 | 6.106 | 1.821 |
|  | A080 | 5.806 | 1.698 |
|  | A103 | 5.488 | 1.597 |
|  | A108 | 4.386 | 1.642 |
| XEC2 | A013 | 5.774 | 1.768 |
|  | A016 | 4.735 | 1.642 |
|  | A032 | 4.883 | 1.615 |
|  | A128 | 5.450 | 1.881 |
|  | A133 | 4.241 | 1.408 |
|  | A141 | 6.557 | 1.696 |
| XEC3 | A035 | 4.606 | 1.639 |
|  | A039 | 5.273 | 1.913 |
|  | A043 | 6.630 | 1.861 |
|  | A047 | 6.961 | 1.866 |
|  | A085 | 4.764 | 1.592 |
|  | A156 | 5.080 | 1.657 |
| XEC4 | A019 | 5.477 | 1.746 |
|  | A026 | 7.332 | 2.201 |
|  | A030 | 7.161 | 2.202 |
|  | A106 | 7.814 | 2.085 |
|  | A118 | 6.952 | 2.105 |
|  | A148 | 6.109 | 1.559 |
| XEC5 | A050 | 5.841 | 1.317 |
|  | A109 | 6.772 | 1.587 |
|  | A122 | 7.505 | 1.630 |
|  | A129 | 5.533 | 1.537 |
|  | A139 | 6.876 | 2.031 |
|  | A140 | 7.629 | 1.515 |
| XEC6 | A034 | 3.393 | 1.378 |
|  | A041 | 4.888 | 1.466 |
|  | A060 | 3.897 | 1.382 |
|  | A107 | 4.759 | 1.609 |
|  | A142 | 3.924 | 1.235 |
|  | A158 | 4.022 | 1.406 |
| XEC7 | A040 | 4.730 | 1.354 |
|  | A083 | 3.933 | 1.499 |
|  | A097 | 6.297 | 1.698 |
|  | A105 | 5.505 | 1.435 |
|  | A110 | 5.362 | 1.540 |
|  | A119 | 5.917 | 1.557 |
| XEC8 | A023 | 6.695 | 1.802 |
|  | A096 | 4.537 | 1.685 |
|  | A113 | 5.169 | 1.499 |
|  | A115 | 6.290 | 1.815 |
|  | A134 | 5.056 | 1.712 |
|  | A153 | 7.475 | 2.385 |
| XEC9 | A018 | 7.722 | 1.962 |
|  | A078 | 7.403 | 1.970 |
|  | A081 | 6.294 | 1.533 |
|  | A104 | 5.410 | 1.474 |
|  | A137 | 6.166 | 1.876 |
|  | A152 | 7.503 | 2.040 |

**Table S5. Raw data of FBG in the validation experiment (mmol/L, n=8)**

| Group | Mice No. | Time | | | | |
| --- | --- | --- | --- | --- | --- | --- |
|  |  | Day 0 | Day 7 | Day 14 | Day 21 | Day 28 |
| NCV | B01 | 3.6 | 3.7 | 3.8 | 3.9 | 3.4 |
|  | B02 | 3.2 | 3.6 | 3.6 | 3.7 | 3.7 |
|  | B03 | 3.9 | 3.7 | 4.0 | 3.7 | 4.0 |
|  | B04 | 4.1 | 3.5 | 3.9 | 3.6 | 4.0 |
|  | B05 | 3.7 | 3.7 | 4.0 | 3.5 | 4.1 |
|  | B06 | 3.5 | 3.6 | 3.4 | 3.8 | 3.6 |
|  | B07 | 3.7 | 3.4 | 3.8 | 3.9 | 3.5 |
|  | B08 | 4.1 | 4.2 | 4.2 | 3.9 | 4.0 |
| DCV | B10 | 13.8 | 14.8 | 13.8 | 14.4 | 14.5 |
|  | B19 | 18.2 | 17.0 | 17.4 | 17.6 | 17.2 |
|  | B20 | 16.5 | 16.6 | 16.3 | 16.6 | 18.1 |
|  | B21 | 17.3 | 15.6 | 17.3 | 17.7 | 15.2 |
|  | B23 | 14.9 | 16.4 | 16.5 | 15.5 | 16.7 |
|  | B29 | 15.6 | 15.7 | 16.0 | 17.4 | 17.5 |
|  | B37 | 19.6 | 19.8 | 18.6 | 18.2 | 18.3 |
|  | B41 | 15.7 | 16.4 | 14.4 | 16.1 | 16.5 |
| METV | B15 | 13.7 | 12.2 | 9.7 | 8.6 | 7.1 |
|  | B25 | 18.7 | 15.4 | 13.3 | 12.5 | 11.1 |
|  | B28 | 13.7 | 13.9 | 10.3 | 9.1 | 7.3 |
|  | B32 | 16.3 | 14.6 | 13.2 | 12.3 | 10.8 |
|  | B35 | 17.4 | 15.6 | 14.3 | 13.0 | 11.4 |
|  | B40 | 16.7 | 16.0 | 12.1 | 11.9 | 9.9 |
|  | B43 | 15.8 | 15.2 | 12.5 | 10.9 | 8.1 |
|  | B45 | 16.9 | 14.5 | 11.2 | 11.0 | 8.7 |
| XECV | B11 | 18.6 | 18.2 | 13.9 | 13.9 | 11.6 |
|  | B13 | 15.4 | 14.2 | 12.0 | 10.4 | 9.4 |
|  | B16 | 16.7 | 15.1 | 13.0 | 10.2 | 8.7 |
|  | B22 | 16.0 | 14.6 | 12.0 | 10.8 | 8.3 |
|  | B24 | 13.0 | 13.8 | 10.5 | 10.0 | 7.9 |
|  | B33 | 14.8 | 16.2 | 13.4 | 11.1 | 8.0 |
|  | B46 | 14.6 | 14.0 | 10.1 | 9.1 | 6.7 |
|  | B48 | 17.8 | 16.7 | 14.6 | 12.1 | 9.5 |

**Table S6. Raw data of OGTT in the validation experiment (n=8)**

| Group | Mice No. | Blood glucose (mmol/L) | | | |
| --- | --- | --- | --- | --- | --- |
|  |  | 0 h | 0.5 h | 1 h | 2 h |
| NC | B01 | 3.4 | 8.8 | 6.8 | 4.6 |
|  | B02 | 4.0 | 11.0 | 7.4 | 5.0 |
|  | B03 | 3.7 | 10.1 | 7.5 | 4.8 |
|  | B04 | 3.5 | 10.7 | 8.4 | 5.2 |
|  | B05 | 3.6 | 10.5 | 7.7 | 5.1 |
|  | B06 | 3.8 | 9.2 | 7.0 | 4.8 |
|  | B07 | 4.1 | 10.1 | 7.9 | 3.5 |
|  | B08 | 4.1 | 9.8 | 6.4 | 5.0 |
| DC | B10 | 13.3 | 22.5 | 27.1 | 21.9 |
|  | B19 | 18.1 | 27.5 | 31.3 | 25.9 |
|  | B20 | 16.7 | 23.9 | 28.1 | 24.6 |
|  | B21 | 17.7 | 25.7 | 29.1 | 25.7 |
|  | B23 | 17.8 | 26.4 | 31.2 | 26.2 |
|  | B29 | 16.1 | 25.2 | 28.8 | 25.1 |
|  | B37 | 18.8 | 28.8 | 32.3 | 26.7 |
|  | B41 | 15.9 | 24.1 | 27.2 | 24.2 |
| METV | B15 | 7.5 | 17.1 | 11.6 | 10.0 |
|  | B25 | 12.6 | 21.4 | 15.0 | 12.9 |
|  | B28 | 8.8 | 16.4 | 14.0 | 8.9 |
|  | B32 | 11.1 | 19.7 | 14.7 | 12.4 |
|  | B35 | 11.6 | 19.9 | 14.3 | 12.4 |
|  | B40 | 9.9 | 20.0 | 10.8 | 12.5 |
|  | B43 | 9.1 | 17.6 | 11.6 | 9.3 |
|  | B45 | 9.7 | 18.2 | 13.1 | 10.9 |
| XECV | B11 | 12.1 | 19.6 | 14.5 | 12.5 |
|  | B13 | 8.5 | 14.7 | 11.9 | 10.3 |
|  | B16 | 9.5 | 18.3 | 12.2 | 10.6 |
|  | B22 | 10.7 | 16.7 | 13.6 | 11.4 |
|  | B24 | 8.8 | 14.2 | 10.7 | 9.6 |
|  | B33 | 9.0 | 15.2 | 11.3 | 9.5 |
|  | B46 | 7.7 | 13.9 | 10.4 | 8.0 |
|  | B48 | 10.4 | 18.5 | 13.1 | 11.0 |

**Table S7. Raw data of HbA1c, TC and TG in the validation experiment (n=8)**

| Group | Mice No. | HbA1c (%) | TC (mmol/L) | TG (mmol/L) |
| --- | --- | --- | --- | --- |
| NC | B01 | 2.8 | 2.939 | 0.873 |
|  | B02 | 3.4 | 2.556 | 1.053 |
|  | B03 | 3.1 | 2.735 | 1.104 |
|  | B04 | 3.4 | 2.736 | 1.004 |
|  | B05 | 3.2 | 2.243 | 1.175 |
|  | B06 | 3.3 | 2.440 | 1.188 |
|  | B07 | 3.5 | 2.588 | 1.287 |
|  | B08 | 3.1 | 2.691 | 0.953 |
| DC | B10 | 6.3 | 6.072 | 2.098 |
|  | B19 | 8.2 | 8.185 | 2.478 |
|  | B20 | 7.7 | 7.056 | 2.354 |
|  | B21 | 6.5 | 7.167 | 2.441 |
|  | B23 | 6.9 | 6.277 | 2.061 |
|  | B29 | 7.9 | 7.492 | 2.421 |
|  | B37 | 8.4 | 8.428 | 2.519 |
|  | B41 | 6.6 | 6.906 | 2.374 |
| METV | B15 | 3.7 | 3.609 | 1.341 |
|  | B25 | 4.2 | 4.367 | 1.489 |
|  | B28 | 3.6 | 3.409 | 1.416 |
|  | B32 | 4.1 | 4.578 | 1.601 |
|  | B35 | 4.4 | 4.897 | 1.594 |
|  | B40 | 3.9 | 4.099 | 1.574 |
|  | B43 | 3.6 | 3.930 | 1.445 |
|  | B45 | 3.9 | 3.821 | 1.577 |
| XECV | B11 | 4.0 | 3.536 | 1.518 |
|  | B13 | 3.7 | 2.853 | 1.330 |
|  | B16 | 3.7 | 2.819 | 1.301 |
|  | B22 | 3.6 | 2.533 | 1.352 |
|  | B24 | 3.3 | 2.365 | 1.270 |
|  | B33 | 3.5 | 2.557 | 1.378 |
|  | B46 | 2.9 | 2.155 | 1.286 |
|  | B48 | 3.9 | 3.054 | 1.390 |
